# Trust and Distrust as Artifacts of Language: A Latent Semantic Approach to Studying Their *Linguistic Correlates*

**DOI:** 10.3389/fpsyg.2020.00561

**Published:** 2020-03-26

**Authors:** David Gefen, Jorge E. Fresneda, Kai R. Larsen

**Affiliations:** ^1^Decision Sciences and MIS Department, LeBow College of Business, Drexel University, Philadelphia, PA, United States; ^2^Marketing, Martin Tuchman School of Management, New Jersey Institute of Technology, Newark, NJ, United States; ^3^Organizational Leadership and Information Analytics, Leeds School of Business, University of Colorado Boulder, Boulder, CO, United States

**Keywords:** trust, distrust, latent semantic analysis, text analysis, machine learning, linguistic correlates

## Abstract

Trust and distrust are crucial aspects of human interaction that determine the nature of many organizational and business contexts. Because of socialization-borne familiarity that people feel about others, trust and distrust can influence people even when they do not know each other. Allowing that some aspects of the social knowledge that is acquired through socialization is also recorded in language through word associations, i.e., *linguistic correlates*, this study shows that known associations of trust and distrust can be extracted from an authoritative text. Moreover, the study shows that such an analysis can even allow a statistical differentiation between trust and distrust—something that survey research has found hard to do. Specifically, measurement items of trust and related constructs that were previously used in survey research along with items reflecting distrust were projected onto a semantic space created out of psychology textbooks. The resulting distance matrix of those items was analyzed by applying covariance-based structural equation modeling. The results confirmed known trust and distrust relationship patterns and allowed measurement of distrust as a distinct construct from trust. The potential of studying trust theory through text analysis is discussed.

## Introduction

### Research Objective

Allowing that socialized knowledge is embedded in the language also through the tendency of words to co-occur together across relevant documents, this study argues that such *linguistic correlates* can reveal much about trust and distrust—key socialization beliefs. That proposition is supported by projecting questionnaire items about trust and distrust and their familiarity antecedent and a behavioral outcome on a semantic space (discussed below) that was built out of a relevant corpus of three psychology textbooks (Myers, 1998), and then analyzing the resulting cosine distance matrix of those questionnaire items. The analysis shows that not only are expected theoretical correlations supported, but also that trust and distrust can be statistically differentiated in this manner—something that survey research using questionnaires had difficulty doing. The ability to mine such knowledge from language may be another tool to study human behavior through text analysis in cases where surveys cannot be given to human subjects, where the context is unknown to them, and where constructs that cannot be easily differentiated such as trust and distrust need to be studied. To clarify, we are not claiming that this method replaces surveys, only that it could complement survey research.

### The Importance of Trust and Distrust in Human Behavior

Interpersonal trust is a key driver of human behavior and a key determinant of interpersonal relationships because it allows people to assume, rightly or not, that they know how those they trust will behave (Blau, 1964; Rotter, 1971; Sztompka, 1999). At the core of trust theory (Luhmann, 1979) is the recognition that people are independent agents who cannot be fully controlled and that these people are not even consistently rational in their behavior. Therefore, contends trust theory, trying to understand how others will behave can introduce so much social uncertainty as to be cognitively overwhelming to the extent that people might refrain from interacting with others they do not trust because they do not understand what is going on. Knowing how the trusted party will behave, i.e., trusting them, allows people to reduce that otherwise overwhelming social complexity to more manageable levels by assuming that the trusted party will behave in expected socially acceptable manners and not in other unexpected socially unacceptable manners (Gefen et al., 2003a).

Because it allows reducing the otherwise overwhelming social complexity to manageable levels, and in doing so allows people to assume that there is a common understanding of what behavior is permitted, interpersonal trust is a key driver of social and economic structures (Williamson, 1985; Fukuyama, 1995; Zak and Knack, 2001). Trust also determines the preference of one vendor or company over another in contracting relationships, again, presumably because the trusting party assumes it knows how the trusted party will behave (Gulati, 1995; Kumar, 1996; Gefen et al., 2008b; Greenberg et al., 2008), and whether any interaction will even occur because when the risk of not knowing what the trusted party will do is too big then people refrain from interacting (Fukuyama, 1995). Because of those reasons, trust is also a key determinant in the adoption of new IT (Gefen, 2004) of many kinds including ecommerce (Gefen et al., 2003b), virtual teams (Jarvenpaa et al., 1998), online communities (Ridings et al., 2002), online software marketplaces (Gefen and Carmel, 2008), online consumer marketplaces such as eBay (Pavlou and Gefen, 2004, 2005; Pavlou and Fygenson, 2006), e-banking (Kaabachi et al., 2017; Ofori et al., 2017), e-government (Warkentin et al., 2018), among others. Trust is even a determinant of susceptibility to phishing (Moody et al., 2017). Basically, trust is a key construct in human behavior (Schoorman et al., 2007).

Trust, as often defined in management papers, is about “the willingness of a party to be vulnerable to the actions of another party based on the expectation that the other will perform a particular action important to the trustor, irrespective of the ability to monitor or control that other party” (Mayer et al., 1995, p. 712). This willingness to trust is based according to Mayer et al. (1995) on beliefs about the *trustworthiness*—ability, benevolence, and integrity—of the trusted party. That assessment of trustworthiness is modeled by Mayer et al. (1995) as the consequence of previous interactions with the trusted party. As research showed, that assessment of trustworthiness can also be the result of the trusting person’s propensity to trust, often modeled as initial trust, that is based on lifelong socialization (Rotter, 1967; McKnight et al., 1998, 2002; Gefen et al., 2003b), a propensity that is influenced *inter alia* by socialization and national culture (Fukuyama, 1995). In the technology context, for ecommerce as an example, this initial trust may be even more important than the perceived usefulness and ease of use of the IT (Gefen et al., 2003a).

Distrust is closely related to trust and is an integral part of trust theory, but it is not just the opposite of trust. Even early on in the study of trust it was recognized that the breakdown of trust results in more than just a reduction in the level of trust in that such a breakdown often results in a transformation of the relationship to one of avoidance (Blau, 1964). Conceptually, distrust is a separate construct entirely from trust (Blau, 1964; Kramer, 1999; McKnight and Choudhury, 2006), dealing with negative beliefs about the other party. Although research based on survey data has found it hard to statistically differentiate between trust and distrust (Benbasat et al., 2008), neuroscience has shown that the neural correlates of trust and distrust are distinctly different (Dimoka, 2010; Riedl et al., 2010b) with trust being mostly associated with neural-correlates that are associated with rewards such as the putamen (the outer part of the lentiform nucleus of the brain) and with information processing such as the dorsolateral prefrontal cortex (DLPFC) while distrust is associated with neural correlates associated with aversion such as the insular cortex and with fear such as the amygdala. Thus, while trust brings people together based mostly on rational reasons, distrust separates them based on fear and aversion. The ability of neuroscience to identify this distinction where survey research could not do so has been one of the reasons suggested for adopting neuroscience into the mainstream of social sciences research (Dimoka et al., 2012). As this study will show, the ability of text analysis to also make this distinction is a point for consideration.

### Trust, Distrust, Familiarity, and the Objective of This Study

A key reason why people trust or distrust, and the context of this study, is because people are socialized into trusting strangers (Rotter, 1971), or a specific group of strangers (Zucker, 1986), or distrusting them as the case might be (Fukuyama, 1995), through socialization and the historical and social information that that socialization conveys (Fukuyama, 1995). In a nutshell, socialization is “learned” familiarity with people at large or with a specific group of people one has not yet encountered. This kind of learning through socialization is typically portrayed as a lifelong experience starting at childhood through education and interaction with other people. People are taught whom to trust and whom to distrust sometimes even on a purely irrational and historically and socially totally irrelevant basis as an integral part of their “education” of learned prejudices and “truisms”^1^.

Across business contexts, familiarity is a significant predictor of trust. Being familiar with the trusted party means that the trusting party knows better what to expect, what the rules of conduct are, how the trusted party might react, and has a reasonable idea of the trusted party’s integrity, benevolence (or at least caring), and capability based on past performance. Being familiar with the other party taps into many of the reasons why trust is needed: being able to assess the trustworthiness of the trusted party as a way of reducing risk (Mayer et al., 1995), being able to better understand what is happening and plan and respond accordingly (Luhmann, 1979, 1988; Gefen et al., 2003b), as well as reducing distrust across social group boundaries (Gefen and Ridings, 2003).

Indeed, choosing a familiar party to contract with can be so compelling an argument that often people will prefer to contract with a party they are familiar with regardless of the price (Gefen and Carmel, 2008). This is not just that trusted vendors can charge a price premium (Ba and Pavlou, 2002). It is that in some cases, specifically low cost contracts to develop software and related services, the trusted party will always win the bid over unfamiliar parties regardless of price (Gefen and Carmel, 2013). And, when price does come into play, such as in large software contracts signed by a bank, then the familiar party will on average be given the contract on terms that require less oversight such as contracting on a time and materials basis rather than a fixed price contract (Gefen et al., 2008b; Benaroch et al., 2016).

Socialization, and the familiarity it creates, is a powerful tool, but not all its teachings are direct and overt. Some of the messages that socialization broadcasts are subtle and hidden in the language we speak. Indeed, as immoral as it may be, the dictionary definition of many words, e.g., racial or social classifications, carry such social praise or stigma that make people feel that they are somewhat “familiar” with the other party based on what they were taught and thus leads them to trust or distrust total strangers based on this socialization. A rather innocuous example is the one Zucker (1986) gives of US banking in the early 1900 where people trusted bankers based on the social class of those bankers who, presumably because one was taught that they belong to a “better” social class, can be trusted. In other words, familiarity can also create distrust. The importance of familiarity in building trust, and by extension reducing distrust, seems to be true across business contexts. This applies in contracting between organizations (Williamson, 1985; Gambetta, 1988; Gulati, 1995; Bolton and Dewatripomt, 2005; Ha and Perks, 2005; Gefen et al., 2008b; Gulati and Sytch, 2008) as well as ecommerce (Gefen, 2000; Pavlou and Fygenson, 2006) and ecommerce recommendation agents (Komiak and Benbasat, 2006), as it is in daily life (Blau, 1964; Luhmann, 2000).

Accordingly, the objective of this study is to argue for linguistic socialization and its implications in a new and expanded context. We argue that *trust and distrust are registered into the very language we speak* and that therefore some aspects of the socialization into trusting and distrusting can be studied through text analysis. To emphasize this registered socialized embedded knowledge, we label it *linguistic correlates*. Technically, it is the same as analyzing how words and vectors of words correlate (or co-appear), expanding on the logic of Gefen and Larsen (2017).

The next sections will show that running text analysis on a semantic space that was built by analyzing a corpus created out of the paragraphs of three psychology textbooks (Myers, 1998)—arguably a reasonable trustworthy repository of theories on human behavior—supports this proposition. This semantic space was chosen because it is accessible in the public domain at lsa.colorado.edu together with an interface that allows projecting combinations of entire sentences on that sematic space. The result of that projection is a matrix of cosine distances that can be extracted for further analysis. That further analysis in covariance-based structural equation modeling (CBSEM) will show that projecting sentences that comprise of survey measurement items dealing with trust, distrust, and related constructs allows the reconstruction of a statistical model based on the cosine distances among each pair of those sentences. And, that in doing so, known psychological relationships of trust and of distrust can be reconstructed.

### Deriving Linguistic Correlates of Trust and Distrust Through a Semantic Space

Just as the conclusions being drawn about sociological events and the interpretation of social constructs will differ based on the sources being read, so too it is recognized that the results of text analysis will depend on the corpus being analyzed and its reliability and connection to the topic being studied. Accordingly, as the study of trust and distrust is clearly in the realm of psychology, and undeniably many other social sciences related to psychology, we chose a semantic space derived from a corpus based on textbooks in psychology.

**FIGURE 1:**
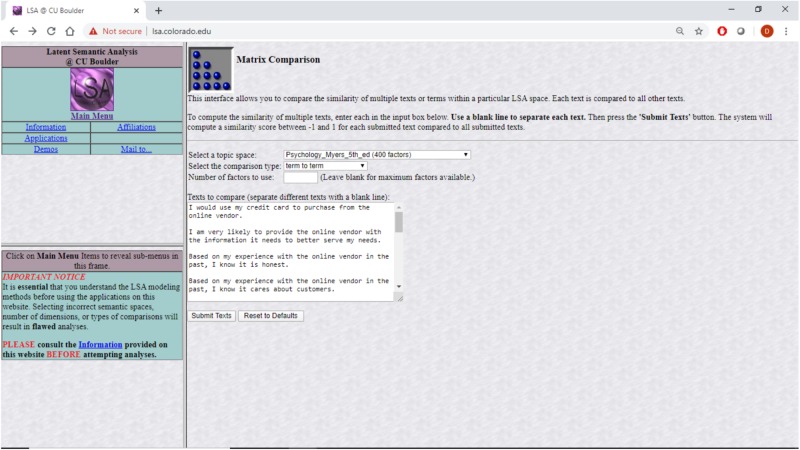
Producing LSA correlations among questionnaire items at lsa.colorado.edu.

The “psychology” semantic space used in this study was created based on a total of 13,902 textbook paragraphs containing 30,119 unique terms. The approach depends on a bag-of-words representation where each paragraph’s word order is abandoned and frequently used terms downweighed before the term-document matrix is subjected to a singular value decomposition (SVD) as described in Larsen and Monarchi (2004). In general practice, 300–500 dimensions are retained (Arnulf et al., 2014). In the creation of this specific semantic space a 398-dimension space was created. This means that each word that is part of one of the textbooks is represented by a 398-dimensional vector of what that term *means* in the context of all the other words. The meaning of a sentence is inferred through the addition of the vectors for each of the words in the sentence, a process known as projection. That sematic space is available in the public domain through an interface at lsa.colorado.edu, shown in Figure 1.

**FIGURE 2:**
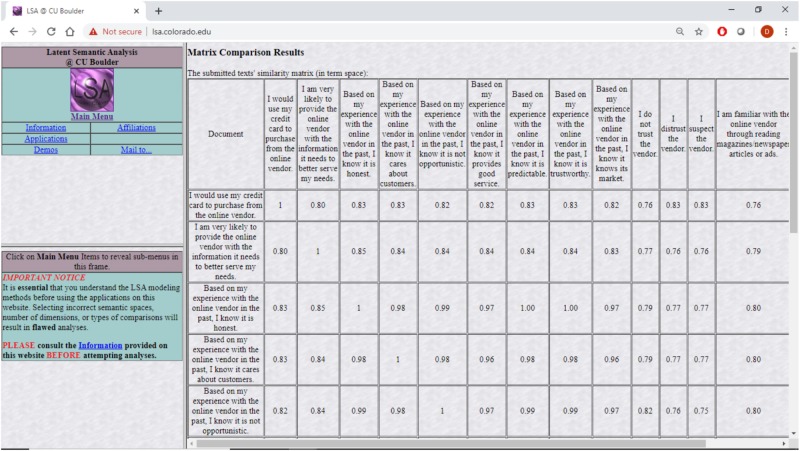
Resulting semantic distances of LSA correlations among questionnaire items at lsa.colorado.edu.

Specifically, survey items from previous research that dealt with trust were projected into this semantic space together with items dealing directly with distrust. The cosine distances among the projected survey items as produced by lsa.colorado.edu were then analyzed using CBSEM. The results discussed in the next sections are as theory predicts. Specifically, the questionnaire items were copied into lsa.colorado.edu, shown in Figure 1, and the derived cosine distances, shown in Figure 2, were then copied and arranged in a matrix form ready to be analyzed with Mplus, shown in Table 1. The questionnaire items appear in Table 2.

**TABLE 1:** Measurement items semantic distance cosines produced by lsa.colorado.edu.

	**USE1**	**USE2**	**TR1**	**TR2**	**TR3**	**TR4**	**TR5**	**TR6**	**TR7**	**DT1**	**DT2**	**DT3**	**FM1**	**FM2**	**FM3**
USE1	1	0.8	0.83	0.83	0.82	0.82	0.83	0.83	0.82	0.76	0.83	0.83	0.76	0.78	0.76
USE2	0.8	1	0.85	0.84	0.84	0.84	0.84	0.84	0.83	0.77	0.76	0.76	0.79	0.8	0.78
TR1	0.83	0.85	1	0.98	0.99	0.97	1	1	0.97	0.79	0.77	0.77	0.8	0.82	0.8
TR2	0.83	0.84	0.98	1	0.98	0.96	0.98	0.98	0.96	0.79	0.77	0.77	0.8	0.82	0.8
TR3	0.82	0.84	0.99	0.98	1	0.97	0.99	0.99	0.97	0.82	0.76	0.75	0.8	0.82	0.8
TR4	0.82	0.84	0.97	0.96	0.97	1	0.97	0.98	0.96	0.78	0.77	0.77	0.8	0.83	0.8
TR5	0.83	0.84	1	0.98	0.99	0.97	1	1	0.97	0.79	0.77	0.76	0.8	0.82	0.8
TR6	0.83	0.84	1	0.98	0.99	0.98	1	1	0.97	0.79	0.77	0.77	0.8	0.82	0.8
TR7	0.82	0.83	0.97	0.96	0.97	0.96	0.97	0.97	1	0.77	0.76	0.75	0.79	0.82	0.79
DT1	0.76	0.77	0.79	0.79	0.82	0.78	0.79	0.79	0.77	1	0.83	0.82	0.75	0.76	0.75
DT2	0.83	0.76	0.77	0.77	0.76	0.77	0.77	0.77	0.76	0.83	1	0.98	0.78	0.78	0.78
DT3	0.83	0.76	0.77	0.77	0.75	0.77	0.76	0.77	0.75	0.82	0.98	1	0.77	0.77	0.77
FM1	0.76	0.79	0.8	0.8	0.8	0.8	0.8	0.8	0.79	0.75	0.78	0.77	1	0.92	0.91
FM2	0.78	0.8	0.82	0.82	0.82	0.83	0.82	0.82	0.82	0.76	0.78	0.77	0.92	1	0.94
FM3	0.76	0.78	0.8	0.8	0.8	0.8	0.8	0.8	0.79	0.75	0.78	0.77	0.91	0.94	1

**TABLE 2:** Measurement items projected on the Myers (1998) textbook semantic space.

**Code**	**Construct/measurement items**	**Standardized loading**
	**Intended Use**	
USE1	I would use my credit card to purchase from the online vendor	0.90***
USE2	I am very likely to provide the online vendor with the information it needs to better serve my needs	0.89***
	**Trust**	
TR1	Based on my experience with the online vendor in the past, I know it is honest	0.99***
TR2	Based on my experience with the online vendor in the past, I know it cares about customers	0.99***
TR3	Based on my experience with the online vendor in the past, I know it is not opportunistic	Dropped
TR4	Based on my experience with the online vendor in the past, I know it provides good service	0.98***
TR5	Based on my experience with the online vendor in the past, I know it is predictable	Dropped
TR6	Based on my experience with the online vendor in the past, I know it is trustworthy	Dropped
TR7	Based on my experience with the online vendor in the past, I know it knows its market	0.98***
	**Distrust**	
DT1	I do not trust the vendor	0.84***
DT2	I distrust the vendor	0.99***
DT3	I suspect the vendor	0.99***
	**Familiarity with the e-Vendor**	
FM1	I am familiar with the online vendor through reading magazines/newspaper articles or ads	0.95***
FM2	I am familiar with the online vendor through visiting the site and searching for CDs/books	0.98***
FM3	I am familiar with the online vendor through purchasing CDs/books at this site	0.96***

***means significant at the 0.001 level.

### The Potential of Studying Linguistic Correlates in the Study of Trust and Distrust

Showing, as this study does, that studying the word associations of trust and distrust produces equivalent results as survey research on trust did, raises the possibility, and clearly more research is needed before such an argument can be made unequivocally, that studying the linguistic registration of trusting behavior in an appropriate source (a textbook on human psychology in this case) might allow new avenues for studying trust and distrust. Such avenues might allow the studying of trust and distrust also in contexts that cannot be studied or do not exist anymore. The context might have changed and the people not available anymore, but at least their study as they are registered linguistically can still be done. This might include studies such as how the meaning and importance of trust and distrust as registered through word associations changed overtime. Given that one cannot administer questionnaires to people who lived in London 150 years ago, but one has easy access to the books written by Charles Dickens and others of that period, such a possibility might open the door to new understandings.

Such an approach to studying trust and distrust—and by extension other constructs, beliefs, attitudes, behaviors, etc.—might also reveal, in a broader context, why non-native speakers of English answer the same questions differently in English versus in their native language, even when the surveys are an exact translation of each other (Harzing, 2005). This approach might potentially also point out possible reasons for social differences about trust and distrust, and provide support for the hypothesized effect of history on trust and distrust as portrayed by Fukuyama (1995). Indeed, comparing the word associations of trust and distrust and the meaning revealed through those in the books of Charles Dickens compared to Henrik Ibsen might be quite revealing.

Moreover, and perhaps this is going on a tangent, if indeed part of our socialization as humans is registered in the language we speak through word correlations, then this might be especially important in predicting how people might understand the role trust and distrust play also in as of yet not quite there technologies. To put this into perspective, research on how we as people trust and distrust others has been about another party that is *human* or composed of a group of people. Specifically, in that past research the trusted party may have been a person [e.g., Blau (1964)], a community [e.g., Ridings et al. (2002)], a market populated by people [e.g., Pavlou and Gefen (2004)], an organization [e.g., Mayer et al. (1995)], a government [e.g., Warkentin et al. (2018)], or a human-like IT interface such as an avatar (Bente et al., 2008; Keeling et al., 2010). But what about a trusted party whose intentions and intelligence are not human or related to people?

Being able to understand, even if only through the knowledge embedded in language, why people trust or distrust in such a case may prove essential with the growing influx of AI into daily lives where AI is creating an environment that is sometimes beyond human understanding, as demonstrated recently in a case of a self-taught AI beating the world champion in *gos* without the world champion even understanding some of the strategies the AI applied (Economist, 2017). The linguistic correlates of trust and distrust might enable modeling human reaction also in such cases of interacting with an AI where the reasons cited above for the importance of trust and distrust do not readily apply. After all, there are no rational assessments of the behavior of an AI agent playing *go*, nor are there considerations of risk, familiarity, social strata considerations, social identification, etc. Nonetheless, being able to model in statistical terms the human response to such a world could be revealing.

The next sections will describe the method we applied to study the linguistic correlates of trust and distrust, why theoretically one might expect there to be linguistic correlates, and some details about the method, and then report the statistical analysis and discuss the results and their potential.

## Materials and Methods

**FIGURE 3:**
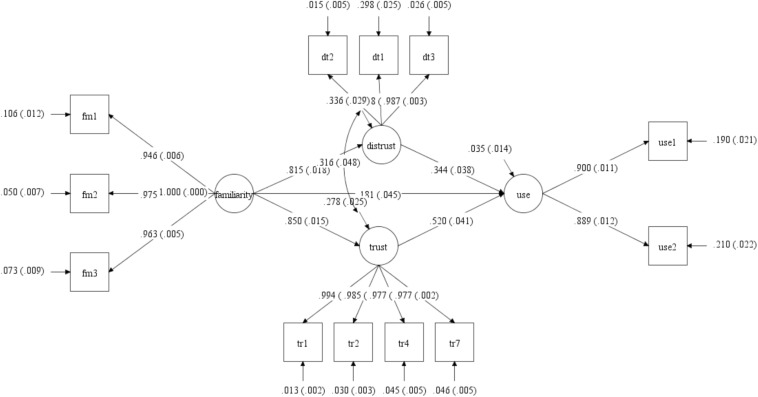
Research model and standardized estimates produced by Mplus.

Replicating the established hypotheses that familiarity builds trust, and adding to it that familiarity may also lead to the opposite, i.e., distrust, as Fukuyama (1995) relates, and further extending into both trust and distrust as major considerations in the decision to purchase online (Gefen, 2000; Dimoka, 2010), the research model is presented in Figure 3. This figure shows the output of the standardized Mplus analysis on the model. Boxes represent the measurement items, which in this case are the questionnaire items that were projected onto the semantic space. These items and their codes appear in Table 2. The covariance among all pairs of those measurement items is constrained in CBSEM so that only the covariance values associated with the paths that are shown in the model as arrows are expressed. All other covariance values are fixed at zero. Fixing those paths to zero frees enough degrees of freedom to include in the model also latent variables, i.e., constructs that while they cannot be measured directly are reflected by the explicit measurement items, as well as how those constructs relate to each other. In this formalization, each measurement item is a function of the latent variable it is assigned to, the circles, and of an error term. For example, fm1, being one of the familiarity measurement items, is predicted by the construct “familiarity” with a path estimate of 0.946 and standard error of 0.006 as well as by a random error term with a path estimate of 0.106 and a standard error of 0.012. The model of the paths leading to the measurement items is known as the measurement model. The paths among the latent variables is known as the structural model. The structural model is what the theory talks about. For example, that trust affects use is shown by the path between the circle labeled trust and the circle labeled use. Those latter paths represent the underlying proposition that the pattern of findings, i.e., supported hypotheses, as revealed in previous survey and archival data research methods can be extracted through linguistic correlates derived from an appropriate corpus.

### Preparing the Model for Study

The model was tested by projecting the [Intended] Use, Trust, and Familiarity scales based on Gefen et al. (2003b) and *ad hoc* items of Distrust on the *psychology* semantic space at lsa.colorado.edu. These questionnaire items are shown in Table 2 with the subsequent Mplus estimated standardized loadings of each item on its related latent variable (construct). The first column contains the item code. This code appears also in Table 1 and in the Mplus code in the Appendix. The second column shows the wording of each item, with a header to make it easier to identify which items relate to which construct. The third column contains the standardized loading of that item on the latent variable, i.e., construct, as produced by the Mplus analysis.

The lsa.colorado.edu site receives as input a set of sentences (or individual words) that are to be projected onto one of several preexisting semantic spaces. See Figure 1. It then builds the cosine distances matrix of each sentence from each other sentence by running a latent semantic analysis (LSA) process. See Figure 2. The process involves projecting each possible pair of sentences as two vectors, each comprising all the words in one of the sentences, on a chosen preexisting sematic space. The idea behind LSA is that words (“terms” in LSA parlance) that tend to appear together have shared dimensions of meaning.

What LSA does is to first create a term to document [frequency] matrix (TDM) of the original corpus, possibly preparing the data beforehand through stemming and other methods, weighing the terms, and then applying SVD to the TDM to reduce the dimensionality of the data (Dumais et al., 1988; Deerwester et al., 1990). It is then assumed that words that appear together on the same principal component (dimension) after this dimensionality reduction exercise share some meaning (Landauer and Dumais, 1997; Landauer et al., 1998). Words can appear in many principal components thus showing the richness of language and that the same word can carry many meanings. The result of the SVD is known as a semantic space. The semantic space analyzed already exists on the lsa.colorado.edu site. The vectors of the sentences can then be projected onto this sematic space, even though the sentences themselves never existed in the original texts. The comparison of these vectors allows a calculation of the cosine distance between them.

At its core, LSA is about word co-occurrences. It is a data-driven approach, and some therefore see it as more objective (Evangelopoulos et al., 2012). As argued, certain words tend to be used together, such as “trust” and “purchase,” so words take on meanings both in terms of the words with which they co-occur, and in terms of words with which they do not co-occur frequently, such as “sky” and “purchase.” Words that co-occur frequently will tend to have a smaller cosine distance between them, and, by extension, two sentences where each contains words that tend to appear in the other sentence will also have a small cosine distance between them. Importantly, LSA works in cases of second and third-level relationships where words do not even need to co-occur, but both co-occur with the same words. For example, LSA will tend to recognize that terms such as “distrust” and “trust” are related even if the words never co-occurred in the text analyzed, for example because both may appear together with the word “transaction” or the word “relationship.”

Because these co-occurrences reflect language used to describe the world, the LSA word vectors contain within them reflections of our shared perceptions of how the world works. Much work has gone into understanding how LSA works relative to the human mind, and Landauer (2007, p. 31) even argued that LSA “demonstrates a computational method by which a major component of language learning and use can be achieved.” The applicability of LSA to partially replicate through text analysis survey responses by people seems to support this contention (e.g., Arnulf et al., 2014, 2018; Gefen and Larsen, 2017). Without entering the debate of what LSA does or does not do [cf., for example, Valle-Lisboa and Mizraji (2007)], we use LSA to address a specific question in a way that is mathematically rigorous and that can be replicated by anyone with an understanding of statistical methods.

More details on how to run LSA in R together with a discussion of the methodological and statistical validity consideration are available at Gefen et al. (2017). As LSA is now widely accepted as a research method, with hundreds of uses within Psychology and Information Systems, we will not go into further depth on the process. Readers interested in this process are referred to one of many detailed descriptions, ranging from mathematical introductions (e.g., Larsen and Monarchi, 2004; Martin and Berry, 2007) to conceptual explanations (e.g. Evangelopoulos et al., 2012; Arnulf et al., 2014).

We chose LSA for several reasons. First, it is an established and tested method and has been so for the last two decades (Tonta and Darvish, 2010; Evangelopoulos et al., 2012). Second, it has been shown to simulate human thought processes, producing survey results that sometimes correspond to how human subjects answer the same questionnaire items (Larsen et al., 2008; Arnulf et al., 2014; Gefen and Larsen, 2017), including assessing the meaning of words through their association with other words (Yeari and van den Broek, 2014; Bhatia, 2017), and even simulating priming effects through word choice (Günther et al., 2016). LSA has even been applied in this context to support the supposition that the meaning of a word is derived through its associations to other words (Kintsch and Mangalath, 2011), and supporting that supposition even by comparing the LSA semantic meaning of a word with eye tracking (Huettig et al., 2006). And, third, the method we apply, running a CBSEM analysis on the correlations derived from LSA semantic spaces has been previously applied to show that the widely supported model of IT adoption, the technology acceptance model (TAM) (Davis, 1989), can be supported by projecting the existing scales of that model on a semantic space that was created out of unrelated newspaper articles (Gefen and Larsen, 2017).

### The Underlying Idea Behind Linguistic Correlates

As specified, the idea being propagated in this study is that socialization knowledge is to some extent ingrained in the language that we speak and write. And that this applies also in word co-occurrence relationships. As a result of this engraining, analyzing word co-occurrence relationships in relevant text could reveal some of that socialization knowledge. Such an argument is supported by the significant and consistent replication of the relationships between the perceived usefulness and the perceived ease of use scales of TAM (Davis, 1989) in both the measurement model (how items load significantly only on their assigned constructs and not on other constructs) and the correlation between the constructs in the structural model by projecting its questionnaire items on two newspaper semantic spaces (Gefen and Larsen, 2017).

The argument for ingrained knowledge in language, expanding on the proposition advanced by Gefen and Larsen (2017), is that if certain words or combinations of words tend to occur together, then these co-occurrence tendencies might be registering socialized knowledge linguistically. Thus, for example, if the word “distrust” and the word “avoid” tend to occur together considerably more than “trust” and “avoid” do, while “trust” tends to co-occur often with “purchase” than “distrust” does then this co-occurrence might be registering that people tend to avoid that which they distrust but tend to purchase from those they trust.

This kind of analysis may actually have the potential to reveal self-censored knowledge too, addressing a known problem with questionnaires. It is well-known that people completing surveys, even anonymous ones, consider both what they think the survey administer wants to hear and what they themselves are implying by their answers (Cook and Campbell, 1979; Shadish et al., 2002). Thus, it would be rather hard to elicit honest non-politically correct prejudices because people completing a questionnaire know that expressing such ideas openly is shunned by society, meaning that there is a bias in such data if it is collected through surveys. However, because LSA analyzes also indirect associations among words, it might catch such prejudices. Indeed, indirect associations of terms identified by LSA has been shown to be beneficial in the case of analyzing medical records to reveal important patterns in the population being studied (Gefen et al., 2018) as well as how IT design battles evolve in the press (Miller et al., 2018). Moreover, terms that are not easily distinguished from each other in the statistical analysis of survey questionnaire items filled by people, might nonetheless be differentiated in text analysis because they each have their own distinct associations with other terms. This differentiation will indeed be shown in the next section.

This is not an argument for causation. It does not mean that people behave as they do because of that linguistically ingrained knowledge, as implied in the “Sapir-Whorf hypothesis” (Hill and Mannheim, 1992) that language determines thoughts and behavior or in an Orwellian control of thought through a *newspeak* language (Orwell, 1948). Rather, the argument is for correlation. People behave as they do for a myriad of reasons, and the language they and others use reflects those tendencies. It may be that their behavior—and more accurately in this case their story-telling about their behavior—reflects their socialization through language, but it may just as well be that language registers the shared aspects of theirs’s and many others’ story-telling.

## Analysis Results

### Analysis Process

The measurement items’ cosine matrix produced by lsa.colorado.edu was entered as input to Mplus version 7.4 and analyzed as a reflective CBSEM. In our measurement model, the reflective CBSEM measurement items are modeled as reflecting a latent variable, known otherwise as a construct. Thus, DT1, DT2, and DT3 all reflect the latent variable (construct) Distrust, and no other construct, while USE1 and USE2 reflect the latent variable Use, and no other, etc. If there are significant cross-loadings, i.e., a loading of a measurement item on a construct it was not assigned to, then CBSEM will identify that cross-loading in the modification index table together with an assumed χ^2^ improvement as well as a noticeable change in the overall fit indices of the model. The measurement model part of a CBSEM model specifies that pattern of measurement items to constructs loadings. The structural model then specifies the relationship among those constructs. Mplus analyzes both the measurement model and the structural model together, highlighting any problems with unspecified covariance or with measurement items whose covariance overlaps. It is standard procedure in CBSEM to drop items that have such problems (SAS, 2013), but it should be reported (Gefen et al., 2011) as we do here.

Items TR5 and TR6 were dropped because the cosine distance between them and between each of them and TR4 was 1.000, meaning that as far as the maximum likelihood algorithm that CBSEM applies as a default for continuous variables these three items are practically indistinguishable from each other. Being indistinguishable from each other, results mathematically in an Mplus observation that “the sample covariance matrix could not be inverted” when those items were included. No other pairs of measurement items had a cosine of 1.000 between them. Item TR3 was dropped to improve model fit (including TR3 did not change the overall model pattern but resulted in an RMSEA of 0.138). It is long established as an acceptable practice to drop items in CBSEM because of such reasons (Bollen, 1989; Jöreskog and Sörbom, 1989).

The Mplus analysis was run specifying that the sample size was 400, which is the rounded number of dimensions created by lsa.colorado.edu for the textbooks when creating the semantic space. As is standard in Mplus for continuous measurement items, we retained the default maximum likelihood analysis. Overall model fit was acceptable (Gefen et al., 2011): χ^2^_48_ = 187.853, RMSEA = 0.085, CFI = 0.985, TLI = 0.979. The Mplus code is available in the Appendix.

### Interpretation of the Analysis

The standardized structural model showed that Use was significantly predicted by Trust (β = 0.52, *p* < 0.001), Distrust (β = 0.18, *p* < 0.001), and Familiarity (Γ = 0.34, *p* < 0.001).^2^ That Trust is a stronger predictor of Use than Familiarity is consistent with anthropological studies where knowing the historical context determines levels of trust and distrust that, in turn, determine behavioral intentions [e.g., Fukuyama (1995)]. These significant predictors of Use are consistent with the literature cited above. Familiarity significantly predicted Trust (Γ = 0.79, *p* < 0.001) and Distrust (Γ = 0.82, *p* < 0.001). This too is consistent with the literature cited above.

The CBSEM model modeled Trust and Distrust as being correlated on account of these two constructs being portrayed in theory as non-overlapping opposite beliefs/assessments of each other with non-overlapping opposite consequences on behavioral intentions (Blau, 1964; Luhmann, 1979; Sztompka, 1999). The theoretical distinction between the Trust and Distrust constructs is also supported by fMRI studies (Dimoka, 2010; Riedl et al., 2010b). The distinction between Trust and Distrust as separate constructs is supported in the CBSEM model through the very low modification index values among the items of the Trust and Distrust constructs. Trust and Distrust as constructs are significantly correlated (θ = 0.32, *p* < 0.001).

R^2^ values were 0.97 for Use, 0.72 for Trust, and 0.66 for Distrust. Cross-loadings were low, as also indicated through the acceptable levels of the RMSEA statistic. Notice that LSA does not specify the sign (plus or minus) of the cosine distances. Hence, the Mplus model shows that the relationships between Distrust and all the other constructs are positive. That is a known limitation of LSA in that it measures the semantic closeness of words, or vectors of words such as the entire sentences of a questionnaire item, as an angle but where the direction of that angle is immaterial.

### Ad Hoc Analysis

As an additional *ad hoc* analysis to establish that differentiating between Trust and Distrust indeed produces a significantly better model, a model that unites these two constructs was compared with the original model. Specifically, the χ^2^ of the original model (χ^2^_48_ = 187.853) was compared with the χ^2^ of an alternative model in which Trust and Distrust were united into one construct. The resulting χ^2^ of this alternative model (χ^2^_51_ = 1073.722) was significantly worse (Δχ^2^_3_ = 855.869), showing that separating Trust and Distrust produces a significantly better model.

## Discussion

### Summary of the Results

The proposition advanced in this study was that socialized knowledge is also ingrained in language, and that this registered knowledge can be extracted through text analysis tools such as LSA and subsequent statistical analysis. These linguistic correlates, as we call them, can be analyzed to both reconstruct existing hypotheses, and do so purely through text analysis and without resorting to distributing surveys to human subjects, as well as be applied to additional analyses not easily performed through survey research. This proposition was demonstrated in the context of studying trust and distrust as they relate to familiarity as an antecedent and to purchase (labeled “use” in other studies) as an outcome.

The analysis supports this proposition, but also highlights some text analysis nuances that should be considered. The analysis shows that linguistic correlates can be analyzed to support the measurement model, showing that the cosine distances between pairs of questionnaire items that are projected on a relevant semantic space can then be analyzed through CBSEM to support the expected significant loadings of those questionnaire items on the latent variable they theoretically reflect. The linguistic correlates also enabled the statistical differentiation between trust and distrust (see *ad hoc* analysis in section “Interpretation of the Analysis”), which has been hard to do with survey research (Gefen et al., 2008a) even though this distinction is suggested in theory (e.g., Fukuyama, 1995; Blau, 1964) and has been shown in neural science (e.g., Dimoka, 2010; Riedl et al., 2010b). The analysis also supports the next part of the proposition that the correlation patterns among those constructs, i.e., the structural model, are consistent with theory. The analysis, however, also shows that the cosine distance between some pairs of items was 1.000, i.e., a perfect overlap, producing a result that is seldom seen in data collected through surveys administered to human subjects, and requiring dropping items accordingly.

The conclusion is that some aspects of socialized knowledge about trust and distrust are ingrained in the language we speak, and that that the registration of this socialized knowledge can be extracted through linguistic correlates to the extent that allows recreating relationships that theory implies.

### Implications for Trust Theory and the Possible Role of Linguistic Correlates

Trust theory and the English language clearly differentiate between trust and distrust, showing that although the two terms are related in their contexts, they are not the same and do not even overlap in their meaning. Such a difference is shown also in this study where both trust and distrust are correlated to familiarity and to use as well as to each other, but their items significantly do not reflect the same, one, latent construct. That studying linguistic correlates could show that difference when survey research that analyzes human subjects’ responses to questionnaires could not, and thereby possibly creating a misinterpretation that trust and distrust overlap in meaning, shows a potential contribution for analyzing linguistic correlates, or at least that linguistic correlates can add significantly to knowledge acquired through survey research.

More specifically from a trust theory perspective, that Trust had a stronger standardized effect on Use (β = 0.52, *p* < 0.001) than Familiarity (Γ = 0.18, *p* < 0.001) did, suggests that, as previous models [e.g., Gefen (2000)] show, it is mainly that familiarity builds trust and that it is mostly trust rather than familiarity that determines behavior. Extending that line of logic, that the standardized effect of Trust is considerably stronger than that of Distrust (β = 0.34, *p* < 0.001) suggests that trust is more important in determining behavior than distrust is in the context of providing information online (see wording of the USE1 and USE2 items) as projected on this specific semantic space. Likewise, that Familiarity affects both Trust (Γ = 0.85, *p* < 0.001) and Distrust (Γ = 0.82, *p* < 0.001) with an almost equal standardized coefficient and that those coefficients are considerably higher than the standardized correlation between Trust and Distrust (θ = 0.32, *p* < 0.001), suggests that familiarity affects trust and distrust through two mostly unrelated channels. Such an observation is consistent with how Fukuyama (1995) describes the evolution of trust and of distrust in different cultures differently based on their histories. What builds trust is not what creates distrust.

Such an ability to differentiate between trust and distrust was brought a decade ago by the burgeoning NeuroIS discipline. (NeuroIS is a name given to the discipline and society that studies neuroscience as applied to information systems). NeuroIS used that same need to differentiate between trust and distrust (e.g., Dimoka, 2010; Riedl et al., 2010b). NeuroIS then used that verification of the trust-distrust distinction through neural correlates to argue that because neuroscience could do so while questionnaire data research could not, to advance a key argument for the importance of such neuroscience research (Riedl et al., 2010a; Dimoka et al., 2012). The same argument may be applicable to text analysis and to linguistic correlates too. Not only can the study of linguistic correlates support behavioral hypotheses through the patterns of word co-occurrences, but it can even support hypotheses that survey data may not be able to. Neuroscience and text analysis are clearly not the same and they undeniably measure different data. Nonetheless, building on that same argument about the ability to study if two constructs might not be the same even when survey research cannot show it, text analysis does have the advantage over neuroscience in that it is cheaper and faster. There are potentially many other such constructs of interest that could be studied.

### Broader Implications for Text Analysis in View of Linguistic Correlates

As Gefen and Larsen (2017) previously suggested, analyzing linguistic correlates may also add another tool to the toolbox that social scientists apply to assess, and maybe statistically control for, priming (Cook and Campbell, 1979), and the inevitable introduction of common method variance in data collected by surveys (Podsakoff et al., 2003; Malhotra et al., 2006). Moreover, text analysis, even if its results do not fully overlap survey analysis given to live subjects, may also provide a cheaper option to pretest existing questionnaires before embarking on a more costly data collection endeavor with subjects. To that, this study adds also the ability to statistically show the discriminant validity, i.e., to differentiate, between constructs that theoretically and linguistically are not the same, but that survey research has not been able to show their discriminant validity.

Moreover, this kind of a method might be especially applicable to the study of contexts that cannot be studied by surveys, such as those unrelated to current actual experiences. Studying linguistic correlates might allow a glimpse into how people in the past thought, and, hence, how concepts of interest changed in their linguistic meaning and associations over time. Clearly talking to actual people or studying actual responses to surveys has its advantages, but there is no known current technology that allows us to ask Charles Dickens or Henrik Ibsen about their take on trust. Studying their writings is an obvious alternative. This method allows doing so semi-automatically. Likewise, such a method could allow studying how these linguistic correlates changed over time by comparing current literature with that of the past.

The comparison of linguistic correlates might also reveal hints as to why, as the Introduction brought, non-native speakers of English answer the same questions differently in English compared to answering the surveys in their native languages, even when the surveys are an exact translation of each other (Harzing, 2005). It may well be that part of the answer is that the linguistic correlates of the constructs being studied in those surveys differ across languages.

Studying linguistic correlates might also reveal partially how people in the present might respond to technologies of the future. That is, studying linguistic correlates could provide a partial picture of the socialized knowledge embedded in the language aspect of why people do what they do. It might be impossible to study how people will react to new technologies such as new aspects of AI that are not available yet—and why in the context of this study they may trust or distrust those—but, looking into people’s linguistic correlates might reveal at least the socialized knowledge embedded language aspect of that question. It might also reveal some hints as to why some cultures might be more open than others to accepting and trusting such AI. Such a glimpse could be of much importance considering that current theories about trust are geared at a person, group of people, or an anthropomorphized party. Current theories of trust address such a target by discussing reasons such as controlling risk and understanding the social environment. It is questionable if and how any of those reasons might apply to an AI. Studying linguistic correlates might at least identify possible motivations and drives that are socialized into language. This also suggests an avenue for possible future research into why people might trust or distrust even when the reasons provided by current research, such as controlling risk (Mayer et al., 1995) or simplifying the social environment to manageable levels (Luhmann, 1979; Gefen et al., 2003b), clearly do not apply. Possibly, such a study of trust and distrust through language usage patterns as revealed through text analysis of a reasonably expert source such as textbooks may allow assessing how people might trust and distrust also in contexts that are beyond their ability to assess risks in or to understand.

### Limitations

The study demonstrated the linguistic correlates proposition through an admittedly simple model. But the very fact that the model could be replicated at all suggests that indeed at least some aspects of social knowledge are recorded in language through the association of words. Presumably, as discussed above, this ingrained knowledge corresponds to how people think either because they learned or socialized that language embedded knowledge or because that language embedded knowledge recorded how people behave. Obviously, replication with other relevant corpora is necessary, but that the analysis supported the proposition is revealing.

Limitations that apply to CBSEM would apply to this method too. Had the model been too complex then the “noise” of covariances that are not included in the model would eventually result in overall poor fit indices. Likewise, many of the overall fit indices, such as χ^2^ and RMSEA are negatively affected as the sample size increases. As the tendency in LSA is to have about 300 to 500 dimensions, and therefore the analysis would be modeled as a sample size of between 300 and 500 data points, the risk of having overall fit indices that do not match the criteria we apply to survey research may become an issue.

Likewise, as with other types of data collection, it is imperative that the source of data be a reliable, valid, and relevant one. This applies in this context much as it does to interviewing experts or giving out surveys. Choosing the correct population (or corpus in this case) is crucial.

Possibly, the limitation that most limits this study and others like it is that the semantic distance, a cosine distance in this case, signifies the strength of the relationship but not its direction, i.e., whether the relationship is positive or negative. Thus, the path from Distrust to Use is positive while according to theory it should be negative. The current method does not address this. Refinements are needed to add a sign value to the cosine values produced by LSA or any other text analysis method that is applied to extract semantic distances.

## Conclusion

This study demonstrated the ability to apply LSA and CBSEM combined to investigate the linguistic correlates of trust and distrust. The study also showed that analyzing linguistic correlates can be applied to differentiate between trust and distrust—something survey research had difficulty in doing. Clearly, the concept of linguistic correlates and the potential of modeling their role in human decision making, is not limited to trust and distrust alone. Nor is this potential limited to the study of only the present. Texts of the past could be just as readily analyzed in the method demonstrated in this paper, opening through linguistic correlates a view to the past and how people in long gone periods might have thought. Practically, this also opens the window to the possible study of how we as people of the present might respond to future technologies and contexts based on our current linguistic correlates.

## Data Availability Statement

The datasets generated for this study are available on request to the corresponding author.

## Author Contributions

DG initiated the idea, built the theory section, ran the CBSEM analyses, and led the project and write-up. JF contributed to the LSA interpretation and to the discussion and write-up. KL ran the LSA analyses, and contributed the discussion and LSA write-up.

## Conflict of Interest

The authors declare that the research was conducted in the absence of any commercial or financial relationships that could be construed as a potential conflict of interest.

## Figures and Tables

**Table:** 

TITLE:	Familiarity to Trust and Distrust to Use based on Psychology textbook
DATA:	FILE IS t2.txt;
	Type=fullcorr;
	Nobs=400;
VARIABLE:	NAMES ARE
Use1 Use2	
TR1-TR7	
FM1-FM3	
DT1 DT2 DT3;	
usev Use1 Use2	
TR1 TR2 TR4 TR7	
FM1 FM2 FM3	
DT1 DT2 DT3;	
ANALYSIS:	Estimator=ML;
MODEL:	Familiarity BY FM1-FM3;
	Distrust By DT1 DT2 DT3;
	Trust BY TR1 TR2 TR4 TR7;
	Use BY Use1 Use2;
	USE on Trust Familiarity Distrust;
	Trust Distrust on Familiarity;
	Trust with Distrust;

## Footnotes

1 As a demonstration of this trust-building or distrust-forming socialization process, think of how many times you heard, or maybe even gave, advisory or precautionary sociological “truisms” such as “Don’t talk to strangers” or “What do you expect of (fill in your preferred racial/religious/social/political etc. noun)? They are always right/wrong/racist!.”

2 In CBSEM notation, exogenous (independent) variables (latent constructs) are labeled β, while endogenous (dependent) variables are labeled ξ. Paths between exogenous and endogenous variables are labeled Γ. Paths between endogenous variables are labeled β. Correlation paths between endogenous variables are labeled θ. Thus, because Familiarity is modeled as affecting Trust, Distrust, and Use, all the paths leading out of Familiarity are labeled Γ. The paths between Trust and Use and between Distrust and Use are labeled β, and the correlation between Trust and Distrust is θ.

## Appendix

### Mplus Code

Output: SAMPSTAT modindices stdyx Tech4 CROSSTABS RESIDUAL.
